# Awake Craniotomy in a Child: Assessment of Eligibility with a Simulated Theatre Experience

**DOI:** 10.1155/2020/6902075

**Published:** 2020-07-05

**Authors:** Jason Labuschagne, Clover-Ann Lee, Denis Mutyaba, Tatenda Mbanje, Cynthia Sibanda

**Affiliations:** ^1^Department of Neurosurgery, University of the Witwatersrand, Johannesburg, South Africa; ^2^Department of Paediatric Neurosurgery, Nelson Mandela Children's Hospital, Johannesburg, South Africa; ^3^Department of Anaesthesiology, University of the Witwatersrand, Johannesburg, South Africa; ^4^Department of Paediatric Anaesthesiology, Nelson Mandela Children's Hospital, Johannesburg, South Africa; ^5^University of the Witwatersrand, Johannesburg, South Africa; ^6^Department of Speech Therapy, Nelson Mandela Children's Hospital, Johannesburg, South Africa

## Abstract

**Background:**

Awake craniotomy is a useful surgical approach to identify and preserve eloquent areas during tumour resection, during surgery for arteriovenous malformation resections and for resective epilepsy surgery. With decreasing age, a child's ability to cooperate and mange an awake craniotomy becomes increasingly relevant. Preoperative screening is essential to identify the child who can undergo the procedure safely. *Case Description.* A 11-year-old female patient presented with a tumour in her right motor cortex, presumed to be a dysembryoplastic neuroepithelial tumour (DNET). We had concerns regarding the feasibility of performing awake surgery in this patient as psychological testing revealed easy distractibility and an inability to follow commands repetitively. We devised a simulated surgical experience to assess her ability to manage such a procedure. During the simulated theatre experience, attempts were made to replicate the actual theatre experience as closely as possible. The patient was dressed in theatre attire and brought into the theatre on a theatre trolley. She was then transferred onto the theatre bed and positioned in the same manner as she would be for the actual surgery. Her head was placed on a horseshoe headrest, and she was made to lie in a semilateral position, as required for the surgery. A blood pressure cuff, pulse oximeter, nasal cannula with oxygen flow, and calf pumps were applied. She was then draped precisely as she would have been for the procedure. Theatre lighting was set as it would be for the surgical case. The application of the monitoring devices, nasal cannula, and draping was meant not only to prepare her for the procedure but to induce a mild degree of stress such that we could assess the child's coping skills and ability to undergo the procedure. The child performed well throughout the simulated run, and surgery was thus offered. An asleep-awake-asleep technique was planned and employed for surgical removal of the tumour. Cortical and subcortical mapping was used to identify the eloquent tissue. Throughout the procedure, the child was cooperative and anxiety free. Follow-up MRI revealed gross total removal of the lesion.

**Conclusion:**

A simulated theatre experience allowed us to accurately determine that this young patient, despite relative contraindications, was indeed eligible for awake surgery. We will continue to use this technique for all our young patients in assessing their eligibility for these procedures.

## 1. Introduction

Awake surgery with direct cortical stimulation is a useful surgical approach to help identify and preserve eloquent areas during cortical and subcortical tumor resections, during surgery for arteriovenous malformations, and for resective epilepsy surgery. Despite extensive literature [1, 2] in the adult population, only a few small case series [3–15] have been published regarding this treatment modality in the paediatric population. With decreasing age, a child's ability to cooperate, understand, and manage the stressful surgical environment of an awake craniotomy becomes more and more relevant. The minimal age for surgery while awake has not been established, and preoperative screening and preparation are essential to identify a cooperative child who can undergo the procedure safely. An appropriate way to address this is to identify the individual level of development and the correlated suitability of the child preoperatively [16].

Here, we describe the use of a simulated theatre experience in order to assess a child's eligibility for an awake procedure.

## 2. Case Report

**Figure 1:**
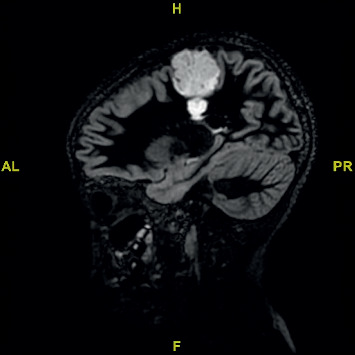
Sagittal flair MRI demonstrating tumour in the primary motor cortex.

**Figure 2:**
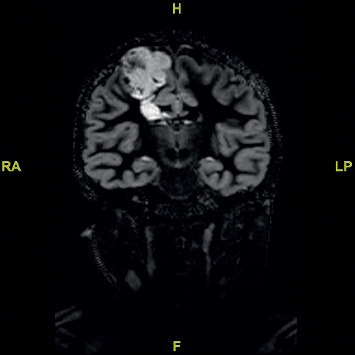
Coronal flair MRI demonstrating tumour in the primary motor cortex.

A 11-year-old female right-handed patient with a long-standing history of drug-resistant epilepsy was referred to our unit for surgical resection of what was presumed to be a dysembryoplastic neuroepithelial tumour (DNET) in her right motor cortex. For the previous 5 years, she had been experiencing 3-4 seizures per month despite appropriate dosages of lamotrigine, valproic acid, and levetiracetam. She had been presented for surgery to another institution, but surgery was denied given the location of the lesion and possible resultant morbidity of surgical resection. Physical examination did not reveal any motor weakness; however, she did have decreased trunk and pelvic control, as well as a lack of co-contraction of her proximal musculature, resulting in poor distal control of her right leg. Furthermore, she was unable to tandem walk, and the occupational therapist felt that at times motor planning was of concern. The physiotherapist found that she became easily distracted and tired and did not like to repeat tasks. MRI imaging revealed a 3.5 cm by 3.5 cm by 5 cm lesion in the right prefrontal cortex, motor strip, with extension into the deep white matter, see Figures 1 and 2. We had several concerns regarding the feasibility of performing awake surgery in this patient, particularly given her young age, her easy distractibility, and inability to follow commands repetitively. We. however, did not want to deny her the benefit of the procedure and therefore devised a simulated surgical experience in an attempt to not only assess her ability to manage such a procedure but also to better prepare her psychologically and emotionally should we decide to go ahead with the procedure.

### 2.1. Protocol of Simulated Theatre Experience

All attempts were made to replicate the actual theatre experience as closely as possible. The patient was dressed in theatre attire and brought into the theatre on a theatre trolley. She was then transferred onto the theatre bed and positioned in the same manner as she would be for the actual surgery. Her head was placed on a horseshoe headrest, but not pinned, and she was made to lie in a semilateral position, as required for the surgery. A blood pressure cuff, pulse oximeter, nasal cannula with oxygen flow, and calf pumps were applied. She was then draped precisely as she would have been for the procedure. Theatre lighting was set as it would be for the surgical case. The application of the monitoring devices, nasal cannula, and draping was meant not only to prepare her for the procedure but to induce a mild degree of stress such that we could assess the child's coping skills and ability to undergo the procedure. The speech therapist spoke her through the procedure, enquiring repeatedly as to what would make her feel more comfortable. She requested music and access to her cell phone to play video games, and these measures were also then provided for on the day of the surgery. Coping skills to deal with intraoperative anxiety and pain were rehearsed at this stage. The surgical team explained to her the steps that she would be put through on the day of surgery. Typical theatre sounds and sights were replicated to further reproduce the stressors she was likely to encounter throughout the actual procedure. The intraoperative motor and language testing protocol was then performed. At the end of the procedure, she was transferred back onto a trolley and transferred to the recovery room. She then visited the Paediatric Intensive Care Unit (PICU). At the end of the mock procedure, a blind vote was cast by the surgical team, anaesthetic team, nursing staff, and allied staff. It was decided that only if all parties involved in the case unanimously voted in favour of performing the case after assessment of her response to the simulation would we proceed with the actual procedure. It was felt all round that she would be able to cope well with the procedure and the vote to continue was unanimous. After seeking the patient's and her parents' input, we decided to schedule the procedure for the following week.

### 2.2. Anaesthesia Protocol

An asleep-awake-asleep technique was planned and employed. On the day of surgery, peripheral intravenous access was obtained after the application of EMLA cream. Anaesthesia was induced and maintained with total intravenous anaesthesia (TIVA) using a propofol target control anaesthesia model (Paedfusor), remifentanil, and dexmedetomidine infusions. A laryngeal mask airway (LMA) was placed, and the patient breathed spontaneously with pressure support provided from the ventilator. The LMA seated well, and ventilation was possible without any leak. A plan for rescuing the airway in case of dislodgement had been made and discussed with the team. The choice of an LMA over an endotracheal tube was in order to avoid any injury occurring to the patient due to coughing on extubation while she was in head pins, and an LMA was easy to resite at the end of the awake phase.

Large bore intravenous access, arterial access, and central venous access were all obtained after the induction of anaesthesia. Standard monitoring including entropy (Entropy, GE Healthcare, Finland) monitoring was used.

With close communication from the surgeon, the propofol and remifentanil infusions were discontinued for the awake part of the craniotomy. At the time of discontinuation of the infusions, in preparation for the wake up, propofol was at an estimated plasma site concentration of 3.2 mcg/ml (dose titrated to clinical effect, entropy, and the neurophysiological monitoring), remifentanil had been running at 0.2 mcg/kg/minute, and the dexmedetomidine had been running at 0.6 mcg/kg/hour. The dexmedetomidine was continued but reduced to a background infusion at a rate of 0.3 mcg/kg/hour for anxiolysis.

The patient opened her eyes, and the LMA was removed 12 minutes after cessation of the propofol and remifentanil. After an initial period of some disorientation, she was able to respond appropriately to questions a further 13 minutes later. The dexmedetomidine infusion was continued throughout the awake phase at 0.1–0.3 mcg/kg/hour. The patient remained calm and cooperative during this time. At the end of the awake period, which lasted a total of 77 minutes, the propofol and remifentanil infusions were recommenced, the patient was anaesthetised, and the LMA was reinserted to maintain airway patency.

### 2.3. Surgical Technique

An asleep-awake-asleep technique was employed. The patient was positioned in a semilateral position. A scalp block, pin site block, and skin incision block were done under general anaesthesia. A Mayfield head clamp was applied, and intraoperative neuronavigation (STEALTH, Medtronic, Minneapolis, MN, USA) was used to assist in locating the brain lesion and to facilitate complete removal. A bone flap was turned, and the dura was infiltrated with local anaesthetic. At this point, the patient was woken up and the LMA removed. When the child was clearly able to communicate with the linguist and was calm and cooperative, the dura was opened. A bipolar electrode, delivering a biphasic current, was applied, and positive and negative motor and speech mapping was performed. For sites involved in language function, an object denomination task, word repetition task, and spontaneous speech were assessed by the speech therapist. During surgical removal of the tumour, subcortical stimulation was used to identify the subcortical motor and language tracts. A threshold of 1 mA was used to limit surgical resection. After gross total tumour resection, the child was anaesthetised, and the LMA was reinserted. Postoperatively, she was woken up and transferred to the PICU. Postoperatively, she displayed very subtle motor weakness in the left leg while walking, but this resolved by discharge. The child was seen six weeks postoperatively. A follow-up MRI scan revealed gross total removal. Histology confirmed a DNET. The patient had not had further seizures, and she had no postoperative deficits. On specific questioning, she reported having no pain or anxiety intraoperatively. She spontaneously volunteered that the mock theatre experience prepared her well for the surgery, and as a result of this, she felt no anxiety. She did not remember the awake part of the procedure well, but could recollect being asked numerous questions and being asked to lie still. She had no significant negative emotions related to the surgery.

## 3. Discussion

Awake surgery with direct cortical stimulation is considered to be the gold standard for identifying eloquent cortical sites in the adult population [2, 17]. Only a few small series, however, have been published regarding this treatment modality in children [3–15].

With decreasing age, the child's ability to cooperate, understand, and manage the stressful surgical environment of an awake craniotomy becomes more and more relevant. [8, 18]. The minimal age for surgery while awake has not been established [19], with some authors [18] consider 11 to 12 years of age being the absolute minimum, whereas other authors have successfully applied this technique in children as young as 8 years of age [6, 7].

An appropriate way to address this is to identify the individual level of development and the correlated suitability of the child preoperatively [16]. The extent and quality of preoperative mental and psychological preparation have an impact on the psychological experience and correlate with the neuropsychological outcome [7, 17, 20, 21], following awake brain surgery.

The standard preoperative preparation for adult awake surgeries includes a detailed explanation of the procedure to the patient and typically some degree of psychological screening and preparation. The tasks to be performed during brain mapping are also typically explained and rehearsed preoperatively [22]. Qualitative studies regarding patients' perceptions and experience of an awake craniotomy along with their satisfaction with the procedure correlate well with their preoperative preparation and trust in the surgical team [23–25].The extent and quality of preoperative mental and psychological preparation have a direct impact on the psychological experience and correlate with the neuropsychological outcome [7, 17, 20, 21],following awake brain surgery. “Adequate patient preparation appears to have a protective effect” [20] from psychological sequelae of an awake craniotomy. According to Girvin [26], “the psychological preparedness of the patient is the most important consideration” for a successful awake craniotomy. Despite this degree of preparation, however, at least 10–15% of adult patients still report severe anxiety during the procedure [27].

Naturally, the preparation of a child for awake surgery is more onerous, firstly to ensure that the child will be cooperative and safe during the awake part of the surgery and also to minimise the anxiety and post-traumatic stress-like symptoms following an awake craniotomy [20]. Riquin et al. [21] describe a very comprehensive preparation phase including having the child examined by a child psychiatrist, hypnotic conditioning of the child, offering the child an opportunity to meet another child who has been operated on while awake, showing the patient pictures and a video describing the atmosphere of the operating room, a visit to the operating room, and a chance to meet the surgical and anaesthetic team.

The use of play therapy to alleviate anxiety and prepare children for surgical procedures is well established, with typical programs including anatomical dolls, videos of surgical procedures, operating room play sets, operating room visits, and familiarisation of the children with theatre equipment [28–31]. Li and Lopez [32] by using a doll demonstration of anaesthesia induction, including obtaining vital signs, providing anaesthetic gas, and obtaining intravenous access and then encouraging the child to perform these procedures themselves on the doll, were able to show a decrease in preoperative and postoperative anxiety levels. Likewise, Armstrong and Aitken [33] tailor their preoperative preparation according to the type of procedure with children being exposed to large bandages, indwelling catheters, etc., as deemed appropriate.

To our knowledge, our unit is the first to report on a complete simulation of an awake craniotomy procedure in order to evaluate a child's ability to undergo such a procedure and furthermore to help mitigate against intraoperative pain and anxiety, thus aiding cooperation and safety during the procedure. We included some of the play therapy and patient preparation measures already described above, but view a simulation run as the natural extension of these measures. As already described, an attempt was made to replicate as closely as possible the actual theatre environment, along with some of the typical stressors encountered while awake during the procedure. This gave us an excellent assessment of the child's ability to cope with the envisioned procedure and confidence in offering the procedure to the child and her parents. Furthermore, by going through the procedure and being able to rehearse coping strategies for pain and anxiety, on the day of the procedure, she was relaxed, completely familiar, and comfortable with the team, theatre, and the processes. Furthermore, the child developed a strong bond with the speech therapist during the simulation run, and this emotional support during the procedure was of enormous benefit in reducing the child's anxiety and no doubt resulting in no reported postoperative psychological symptoms or anxiety.

## 4. Conclusion

Awake surgery with direct cortical stimulation is a useful surgical approach to help identify and preserve eloquent areas during cortical and subcortical tumor resections and should not be denied to children because of an arbitrary minimum age level. Determining the suitability, however, of a patient for an awake procedure in the paediatric population requires significant preoperative assessment and evaluation. We feel employing a simulated theatre experience, mimicking the actual theatre experience as closely as possible, is invaluable in determining a child's eligibility for such a procedure and also serves them well in dealing with the psychological anxiety and potential sequelae of such a procedure.
